# Surveillance of Viral Respiratory Infections within Maximum-Security Prison, Australia

**DOI:** 10.3201/eid3108.240571

**Published:** 2025-08

**Authors:** Nicolas M. Legrand, Rowena A. Bull, Behzad Hajarizadeh, Andrew R. Lloyd, Kirsty Johnston, Katya Issa, Christine Harvey, Alicia Arnott, Dominic E. Dwyer, Vitali Sintchenko, Luke Grant, Gregory J. Dore, John Kaldor, Marianne Martinello

**Affiliations:** University of New South Wales, Sydney, New South Wales, Australia (N.M. Legrand, R.A. Bull, B. Hajarizadeh, A.R. Lloyd, G.J. Dore, J. Kaldor, M. Martinello); St Vincent’s Correctional Health, Sydney (K. Johnston, K. Issa); New South Wales Ministry of Health, Sydney (C. Harvey); New South Wales Health Pathology, Sydney (A. Arnott, D.E. Dwyer, V. Sintchenko); University of Sydney, Sydney (D.E. Dwyer, V. Sintchenko); Corrective Services New South Wales, Sydney (L. Grant).

**Keywords:** Viruses, viral infections, respiratory infections, SARS-CoV-2, COVID-19, incarceration, Australia

## Abstract

Limited surveillance data have hindered understanding of SARS-CoV-2 transmission within prisons. We integrated routine surveillance data with viral sequencing to investigate transmission dynamics and associated factors during a Delta variant outbreak in a maximum-security prison in Sydney, New South Wales, Australia. Infection incidence and associated factors were determined by using person-time and Cox regression. We generated transmission chains by integrating epidemiologic and viral sequencing data. Of 1,562 patients, SARS-CoV-2 infection was diagnosed in 169 (11%), predominantly acquired in prison and asymptomatic. Prisonwide testing identified substantial unrecognized transmission, and 4 subvariants indicated multiple viral introductions. Infection was associated with housing location, having a cellmate (regardless of infection status), and vaccination status. Our findings underscore the inadequacy of symptom-based testing and the efficacy of entry-quarantine, strategic housing, extensive testing, and vaccination in reducing transmission. This integrated approach to surveillance and genomic sequencing offers a valuable model for enhancing infectious disease surveillance in correctional settings.

As the global health community transitions from a pandemic response to managing COVID-19 as an endemic disease, environments such as prisons and other congregate settings continue to demonstrate unique public health challenges. Implementing minimally restrictive preventive measures, such as physical distancing, is difficult because of inherent structural and organizational barriers, including close confinement, poor ventilation, and limited capacity for medical isolation (*1*,*2*). In addition, the continual cycle of custodial transfers, reception, and releases increases the likelihood of infection introduction and the potential for outbreaks of acute respiratory infection, including COVID-19, among incarcerated persons (*3*–*6*).

Substantial knowledge gaps remain regarding factors associated with transmission during acute respiratory infection outbreaks in prisons. Previous studies of COVID-19 outbreaks in prisons were limited by low SARS-CoV-2 testing coverage (*7*–*9*), inconsistent testing schedules (*10*–*12*), and minimal genomic data (*13*–*15*). In addition, the cycle of admissions and departures complicates data completeness, leading to uncertainty around the at-risk population size. Previous research often relied on approximating at-risk population size (*7*,*16*,*17*), which overlooks true variability over time, introduces potential bias because of residual confounding, and affects the quality of time-series analyses. Understanding transmission dynamics in prison is crucial for enhancing effective outbreak response strategies and enabling timely interventions to mitigate risk. Because of the higher prevalence of chronic diseases among incarcerated persons (*18*,*19*), improving systematic approaches to reducing acute respiratory infection (including SARS-CoV-2) outbreaks within prisons and other congregate settings remains a public health concern.

After several months of no local SARS-CoV-2 transmission in Australia, the first case of the Delta variant was confirmed on June 16, 2021, by whole-genome sequencing (WGS) in Sydney, New South Wales. Public health restrictions on movement were enacted on June 26, 2021, because of the evidence of increasing community infection. During this period of increasing community spread, an outbreak of the SARS-CoV-2 Delta variant occurred within prison in Australia, spanning 48 days. The outbreak began 6 months after COVID vaccines were available in Australia (February 22, 2021) and precipitated a multijurisdictional public health response, building upon substantive existing control measures including quarantine on entry, isolation of recognized cases, and personal protective equipment for staff (Appendix Figure 1). The response to the outbreak included a total prison lockdown on day 13, continuous mass surveillance testing, genomic sequencing of SARS-CoV-2 with WGS, and ongoing vaccination. Our study objectives were to determine the incidence of SARS-CoV-2 infection and identify factors associated with transmission among incarcerated persons during a large-scale outbreak.

## Methods

### Study Design and Setting

**Figure 1:**
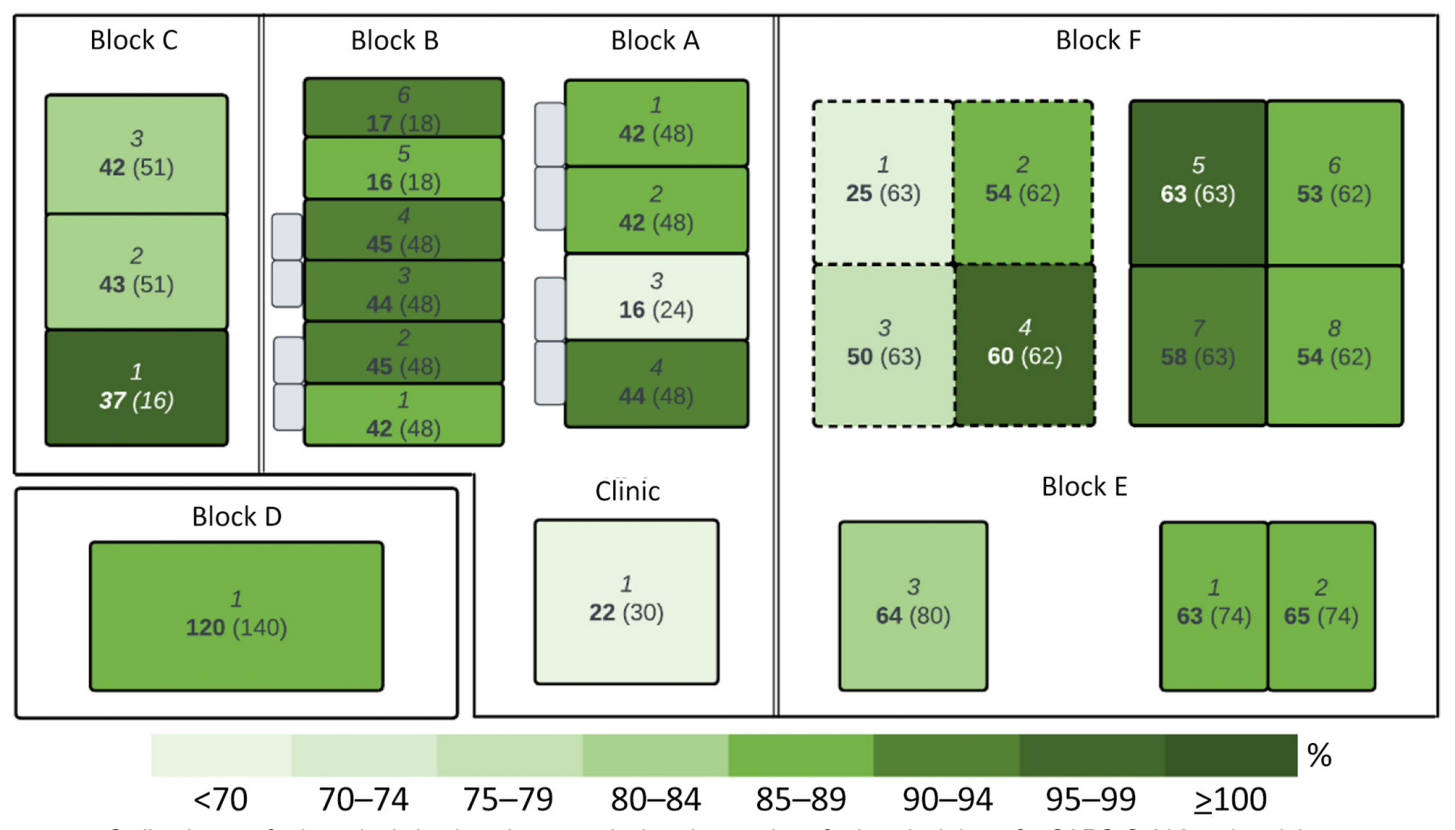
Stylized map of prison depicting housing capacity by wing on day of prison lockdown for SARS-CoV-2 outbreak in a maximum-security prison in Australia, 2021. Blocks A–E are general housing for incarcerated persons not in quarantine or isolation. Block F contained both general housing wings (wings 5–8) and 4 dedicated quarantine wings (wings 1–4) for persons undergoing a mandatory 14-day quarantine period before entry. Block D, the minimum-security wing, was located outside the main prison. Each wing is indicated in italics, the number of incarcerated persons is indicated in bold, and the reported maximum operational capacity is in brackets. The categorical color gradient of each unit indicates percentage of housing capacity. Quarantine zones in block F (1–4) are indicated by dashed outlines. Double lines represent internal walls. Gray shaded areas in blocks A and B represent external yard space, separated by chain-wire fencing. External yards are found in all areas but not displayed in each instance because they are contained within wings and are not considered a potential site of interwing transmission.

This prospective cohort study, conducted in a prison in Sydney, followed the strengthening of reporting of observational studies in epidemiology reporting guidelines for observational cohort studies. The prison had a maximum operational capacity of 1,300 beds, housing men who were sentenced or on remand. The prison was divided into 6 housing units (blocks A–F) and a clinic (Figure 1). Operational housing capacity across blocks ranged from 118 to 500 persons, with 30 beds in the clinic (Figure 1). Housing arrangements were either single or 2-bed occupancy. The study period was 48 days, commencing with the identification of the first COVID-19 case and ending on the date of the last laboratory-confirmed case.

### Participants and Data Sources

We included all persons housed in the prison during the study period (Appendix Figure 2). We used routinely collected person-day–level data gathered by local health authorities and corrective services (sociodemographic characteristics, prison entry and exit, SARS-CoV-2 nucleic acid testing [NAT], vaccination administration, housing location), and SARS-CoV-2 WGS data for analysis. We conducted NAT by using EasyScreen SARS-CoV-2 RT-PCR (Genetic Signatures, https://geneticsignatures.com) or the GeneXpert SARS-CoV-2 assay (Cepheid, https://www.cepheid.com). We conducted prisonwide surveillance SARS-CoV-2 testing, regardless of symptom or contact status, by living unit on a continuous 72-hour basis. All health and prison staff providing direct, in-person care of incarcerated persons were required to wear personal protective equipment appropriate for contact, droplet, and airborne precautions (P2/N95 mask, eye or face protection, gown, gloves) and to assess inmates in their cells.

### Study Definitions and Outcomes

We defined the date of infection by symptom onset or positive NAT, whichever came first. We defined the infectious period of positive cases as starting 2 days before symptom onset or sample collection, whichever came first, and ending 14 days later. We assigned case definitions relative to their potential source of infection (community, prison, or unknown) and the confidence of the source of transmission (probable, possible, unknown) (Appendix). We defined clinical infection severity as asymptomatic, mild, moderate, or severe (*20*). We defined SARS-CoV-2 infection as a positive SARS-CoV-2 NAT result.

### WGS

We sent samples with detectable SARS-CoV-2 RNA to the Institute of Clinical Pathology and Medical Research, New South Wales Health Pathology (Sydney), for WGS to support contact tracing and cluster analysis. We extracted viral genomes from upper respiratory tract swabs and PCR amplified by using the Illumina Midnight (Illumina, https://www.illumina.com) sequencing protocol and sequenced PCR products by using the Illumina platform. We generated a consensus sequence from each sample to conduct genomic sequence comparisons between suspected transmission clusters, as previously described (*21*).

We aligned the consensus genomes by using MAFFT v7.471 (FFT-NS-2, progressive method) (*22*). We manually inspected the consensus genomes and excluded any sequences missing >20% of the genome. We observed poor sequence read coverage across regions 21381 and 21683; therefore, we removed the region from all alignments. We constructed a phylogenetic tree visualizing sequence similarity between different samples by using the maximum-likelihood approach in IQTree v1.6.7 with the general time-reversible with unequal empirical base frequency and proportion of invariable sites substitution model and 1,000 bootstrap replicates (*23*). We defined transmission clusters genomically on the basis of shared mutational profiles and by clustering on the phylogenetic tree (Appendix Figure 3). We considered sequence pairs or clusters sharing <3 mutations genomic evidence in support of direct or recent transmission.

### Statistical and Molecular Analyses

We calculated incidence by using person-time of observation and reported as the number of infections per 100 person-years. We calculated 95% CIs for rates by using a Poisson distribution. Time at-risk commenced on day 1 of the study period, or the date of prison entry for people received later, and was censored at day 48, or at the earliest occurrence of the incarcerated person testing NAT positive, being transferred out before lockdown, or being released.

We used Cox proportional hazards regression analysis to estimate hazard ratios (HRs) and 95% CIs to evaluate factors associated with SARS-CoV-2 transmission, by using person-level time-varying covariates for changes in factors related to exposure. This approach enabled evaluation of hazards on the basis of each person’s status daily, capturing the transition in infection risk status. Those factors were determined a priori and included housing location, vaccination status, and cellmate exposure in the preceding 14 days. We determined the frequency of cellmate exposures over the course of the outbreak by using a moving 14-day window.

We mapped within-prison movement of case-patients and cellmates and generated chains of transmission including direction to the individual level (Appendix). Genomic sequencing was available for 128 (76%) cases, and we used the sequencing data to validate the hypothesized chains of transmission on the basis of epidemiologic data.

We performed statistical analyses by using Stata software version 17 (StataCorp, LLC, https://www.stata.com). We conducted the data visualization by using Microsoft Power Bi (Microsoft, https://www.microsoft.com) and Lucidchart (https://www.lucidchart.com).

### Study Oversight

Because this investigation was a public health priority, it was conducted under the Public Health Act at the request of the NSW Ministry of Health and in collaboration with St Vincent’s Correctional Health NSW, Justice Health and Forensic Mental Health Network, the Institute of Clinical Pathology and Medical Research, and the Kirby Institute for Infection and Immunity in Society. This study received ethical approval from the University of NSW Human Research Ethics Committee (approval no. HC220683). A waiver of consent was granted as the research involved secondary analysis of existing deidentified data collected during routine public health activities.

## Results

### Demographic and Clinical Characteristics

During the 48-day study period, 1,562 persons were housed in the prison, and SARS-CoV-2 infection was diagnosed in 169 (11%) incarcerated persons (Appendix Figure 2). There was complete data capture for each person, covering SARS-CoV-2 NAT, vaccination status, and housing location. Total follow-up time was 131 person years (median follow-up 39 days [IQR 14–48 days]). With prisonwide surveillance testing, 9,575 SARS-CoV-2 NATs were conducted (249 positives, 9,326 negatives; median tests of incarcerated persons = 7 [IQR 3–11]). Of 169 persons with laboratory-confirmed COVID-19 (median age 34 years [IQR 18–78 years]), 62 (37%) were symptomatic at diagnosis, whereas in 122 (72%) patients, symptoms developed at some point during infection. Asymptomatic or mild infections accounted for 93% (n = 157) of cases (Table 1); 7 cases were moderate and 1 was severe.

**Table 1:** Demographic and clinical characteristics of incarcerated persons with SARS-CoV-2 in maximum-security prison, Australia, 2021*

Case characteristics	Value, n = 169
Age group	
18–29	65 (38)
30–39	62 (36)
40–49	26 (17)
50–59	13 (8)
>60	3 (2)
Likely source of infection acquisition	
Prison	153 (92)
Community	16 (8)
Housing location	
General unit	69 (41)
Quarantine unit	100 (59)
Cellmate placement in 14 days before date of infection	
Housed alone	20 (12)
Housed with COVID-19 positive cellmate	47 (28)
Housed with COVID-19 negative cellmate	102 (60)
Duration of incarceration before diagnosis, d	
Median (IQR)	47 (19–100)
Range	0–804
SARS-CoV-2 vaccination status at time of diagnosis	
2 doses, >2 weeks after second dose	8 (5)
2 doses, <2 weeks after second dose	5 (3)
1 dose	45 (27)
0 doses	111 (66)
Disease severity	
Asymptomatic	43 (25)
Mild	114 (68)
Moderate	7 (4)
Severe	1 (1)
Unknown	4 (2)
Symptomatic at diagnosis	62 (37)
Symptomatic ever	122 (72)
Reason for testing	
Mass testing schedule	140 (83)
Entry screening	7 (4)
Quarantine screening	18 (11)
Symptom driven, close contact, or both	4 (2)
SARS-CoV-2 Delta subvariant	
130	35 (21)
130.18	11 (7)
130.34	65 (38)
130.74	17 (10)
Missing	41 (24)

*Values are no. (%) except as indicated. IQR, interquartile range.

COVID-19 vaccination coverage was low at the outbreak’s onset, driven by high refusal rates. At the time of lockdown, 853 (70%) of incarcerated persons had not been vaccinated, 262 (21%) of incarcerated persons had received 1 vaccine dose, and 111 (9%) of incarcerated persons had received 2 doses. By the end of the study period, after the implementation of a vaccination campaign as part of the outbreak response, the percentages of persons vaccinated increased; 121 (13%) of incarcerated persons were not vaccinated, 247 (26%) of incarcerated persons received 1 vaccine dose, and 567 (61%) of incarcerated persons received 2 doses; only 14 incarcerated persons declined vaccination.

### Location and Source of Acquisition

Evidence of SARS-CoV-2 transmission was found in 4 of the 6 housing blocks (A, B, E, and F), as well as in the clinic; 59% (n = 100) of cases occurred within quarantine units. Most SARS-CoV-2 infections were likely acquired in prison (91%, n = 153) and were diagnosed after 14 days of continuous incarceration (n = 144). SARS-CoV-2 was diagnosed in a smaller group (n = 9) within 14 days of prison entry; epidemiologic and genomic evidence, including exposure to a COVID-19 positive cellmate (n = 4) and genomic cluster membership (n = 5), suggested those persons also acquired the infection in prison. Among prison-acquired cases (n = 153), a probable or possible source of transmission was identified in 141 cases (92%) (probable, n = 77; possible, n = 64). Of sequenced cases (76%, n = 128), 4 Delta subvariants were identified: NSW130.0 (n = 35), NSW130.18 (n = 11), NSW130.34 (n = 65), and NSW130.74 (n = 17). The predominant circulating Delta subvariant in NSW at that time was NSW130.0.

### Incidence of SARS-CoV-2 Infection and Factors Associated with Transmission in Prison

Infection incidence was 121 (95% CI 104–142)/100 person-years. SARS-CoV-2 infection was associated with housing type, vaccination status, and cellmate exposure (Table 2; Appendix Figure 4). Incidence was higher in quarantine units compared with general units (aHR 1.90 [95% CI 1.39–2.59]). Incidence was higher among unvaccinated persons, compared with those who received 2 doses (aHR 0.46 [95% CI 0.27–0.79]) of COVID-19 vaccine. Infection risk was highest for persons exposed to a SARS-CoV-2–positive cellmate in the 14 days before SARS-CoV-2 diagnosis (aHR 18.87 [95% CI 10.99–32.37]).

**Table 2:** Incidence rate of and factors associated with SARS-CoV-2 infection in incarcerated persons in maximum-security prison, Australia, 2021*

Characteristics	No. infections	Follow-up time, person-years	Infection incidence/100 person-years (95% CI)†	Unadjusted hazard ratio (95% CI)	Adjusted hazard ratio (95% CI)
Overall	160†	131	121 (104–142)	0.98 (0.97–1.0)	0.99 (0.97–1.01)
Living unit					
General units	70	87	80 (63–102)	Referent	Referent
Quarantine units	90	44	202 (165–249)	2.43 (1.77–3.34)	1.90 (1.39–2.59)
Vaccination status					
Unvaccinated	102	56	183 (151–223)	Referent	Referent
First dose	45	47	95 (71–128)	0.67 (0.45–0.98)	0.84 (0.59–1.19)
Second dose	13	28	45 (26–78)	0.43 (0.24–0·77)	0.46 (0.27–0.79)
Cellmate exposure status					
No cellmate	18	35	51 (33–82)	Referent	Referent
NAT-negative cellmate	95	94	101 (83–124)	1.78 (1.07–2.95)	1.84 (1.11–3.05)
NAT-positive cellmate	47	2	1,888 (1,418–2,153)	21.74 (12.9–36.72)	18.87 (10.99–32.37)

*NAT, nucleic acid testing. †Infection incidence analyses included 160 cases of SARS-CoV-2 infection among incarcerated persons; 9 cases were excluded because of positive test on prison entry (n = 4) or after transfer or release into the community (n = 5).

### Time-Course of Outbreak Detection and Public Health Response

**Figure 2:**
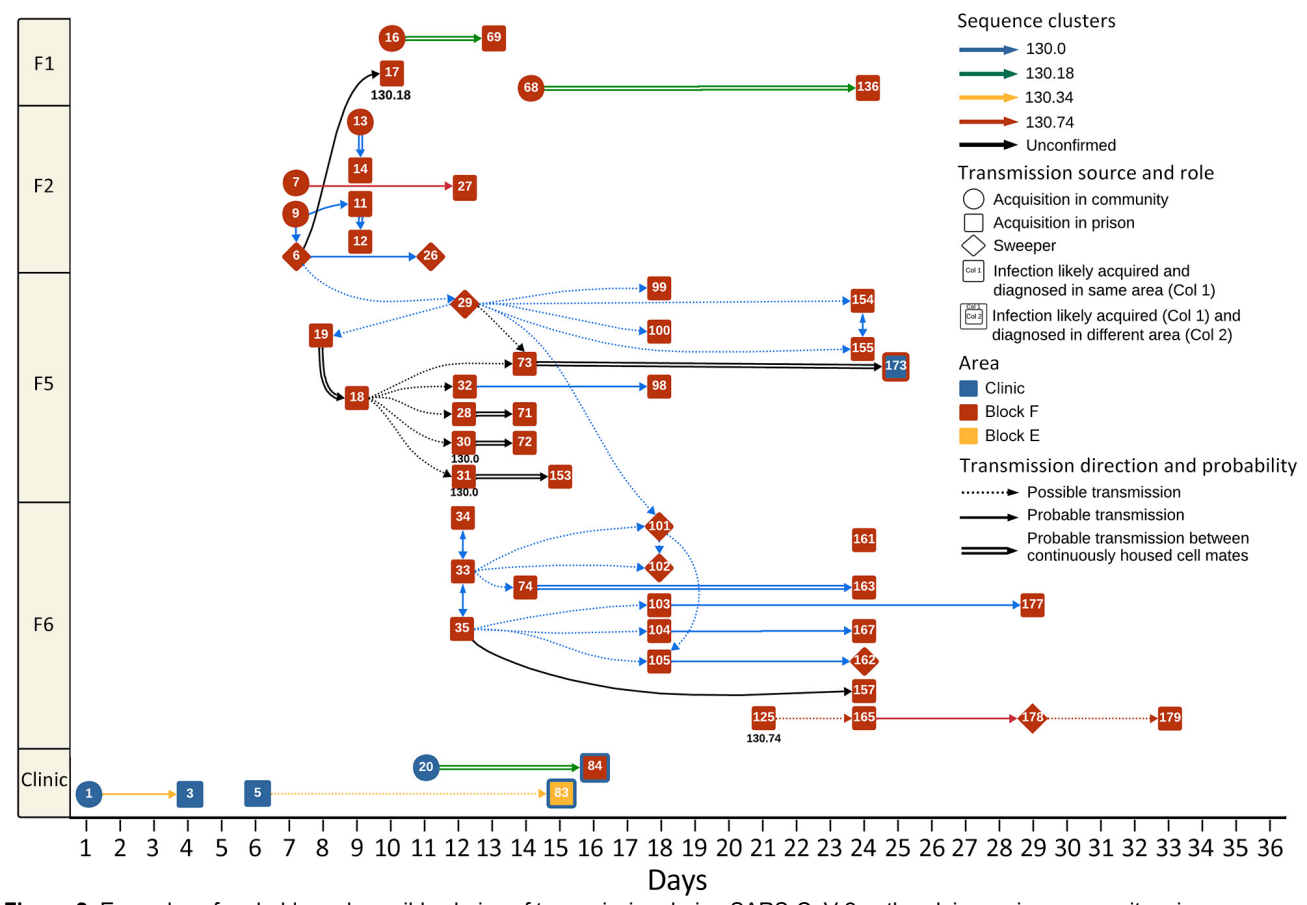
Examples of probable and possible chains of transmission during SARS-CoV-2 outbreak in maximum-security prison in Australia, 2021. Cases are plotted temporally on the basis of infection date and spatially according to both location of infection acquisition and location at the time of diagnosis. Diamonds denote incarcerated persons working as sweepers and circles indicate community-acquired cases. Transmission is visualized with solid lines for probable transmission, dotted lines for possible, and double solid black lines for transmission between cellmates consistently housed together before and after lockdown. Arrowheads mark the likely direction of transmission, and line colors represent genomic sequence clusters. For transmission pathways where only 1 genomic sequence was available, the sequence identification is displayed below the patient.

We plotted the prison transmission chains (Figure 2). SARS-CoV-2 infection was first detected in the prison by a routine entry screening test conducted on an asymptomatic person housed alone. Over the next 5 days, 2 more incarcerated persons (patients 3 and 5), also housed alone in the clinic area and within their 14-day entry quarantine period (negative SARS-CoV-2 NAT on entry), had SARS-CoV-2 diagnosed through routine NAT screening on day 11 of incarceration. Of note, there was no evidence of close contact between those patients, raising concerns about the role of cell proximity and structural drivers in transmission. Genomic sequencing and phylogenetic analysis revealed that those cases belonged to the NSW130.34 cluster, indicating a close genetic relationship and a common source of infection (Figure 2; Appendix Figure 5).

Independent of that cluster, a newly incarcerated person housed within 1 of the prison’s dedicated entry quarantine wings (block F3) returned a day 4 SARS-CoV-2–positive NAT, 2 days into their mandatory 14-day entry quarantine period. Over the next 7 days, 11 additional persons housed in the entry quarantine wings (F1–F4) had SARS-CoV-2 infections diagnosed. Patient 6, an unvaccinated, asymptomatic person whose SARS-CoV-2 infection was diagnosed by surveillance screening and was continuously incarcerated for >14 days, was housed alone in the quarantine wing F2, where they worked as a sweeper, a person whose job involves domestic tasks and has permission to move more freely within the designated area, for 6 weeks. Acquisition likely occurred while patient 6 was undertaking sweeper duties, with supportive evidence provided by WGS (Figure 2; Appendix Figure 5). The movements and interactions of patient 6 with other sweepers likely enabled transmission within F1 and the subsequent spread from quarantine wings F1–F2 to F5–F6 before the facility lockdown on day 13. Epidemiologic and genomic clustering analysis supported the probability that transmission had occurred among blocks F2, F5, and F6, with most sequences belonging to the NSW130.0 cluster (Appendix Figures 3 and 5).

By day 13, SARS-CoV-2 infection was confirmed in 28 persons. In response, the prison was placed into lockdown, confining incarcerated persons to their cells, limiting internal movements to interrupt further transmission, and a coordinated outbreak response was enacted. Key response measures included continual mass surveillance, SARS-CoV-2 testing, clinical isolation of those with SARS-CoV-2 diagnosis and their cellmates, rapid establishment of onsite healthcare provision for persons with COVID-19, contact tracing of incarcerated persons and staff, cessation of new receptions and prison transfers, and scaling up of voluntary vaccination. Healthcare workers underwent SARS-CoV-2 NAT every 3 days before facility entry, and prison staff were tested 3 times weekly. On day 26, the testing protocol transitioned from NAT to rapid antigen testing for all staff screening. Staff with positive or invalid rapid antigen testing results underwent onsite NAT.

**Figure 3:**
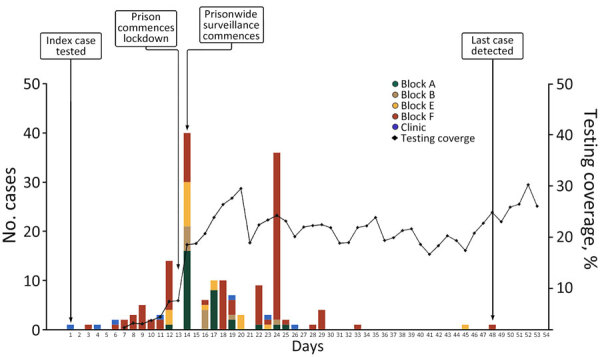
Timeline of SARS-CoV-2 infections during outbreak in maximum-security prison in Australia, 2021. Bars indicate numbers of cases by housing location and date of infection; line indicates 7-day moving average of testing coverage.

Prisonwide SARS-CoV-2 NAT-based surveillance (regardless of symptom or contact status) was initiated the day after lockdown (day 14), and 80% of the total prison population was tested. After lockdown, a structured NAT testing schedule was implemented by living unit, testing all incarcerated persons every 72 hours. Daily 7-day-average testing coverage increased from 2% in the first week to 20% in the second week and thereafter maintained coverage of 19%–25% (Figure 3). The implementation of continual mass testing detected ongoing, previously unrecognized transmission with cases in blocks A, B, E, and F (Figure 3). The outbreak was considered resolved after 14 days had elapsed (1 maximum incubation period) with no new cases.

## Discussion

An outbreak of COVID-19 occurred in a maximum-security prison in Australia, spanning 48 days, with 169 cases of SARS-CoV-2 infection diagnosed. Transmission occurred within wings dedicated to entry-quarantine and areas housing the general population. Infection severity among cases was predominantly asymptomatic or mild. The prison lockdown and mass testing schedule initiated as part of the outbreak response identified many asymptomatic and presymptomatic cases who were not detected through symptom-based testing or reported close contact. Genomic analysis identified distinct genomic clusters involving 4 Delta subvariants, indicating multiple independent viral incursions into the prison. Increased SARS-CoV-2 infection risk was associated with cellmate exposure, unvaccinated status, and housing in units dedicated to quarantine and isolation.

Global investigations of SARS-CoV-2 outbreaks in prisons have underscored the significance of systematic testing schedules and genomic sequencing (*24*,*25*). When implemented, mass testing frequently identifies widespread unrecognized infection and transmission (*26*,*27*). In this study, the most substantial peak in case detection occurred after the first round of mass surveillance testing, conducted the day after lockdown, which resulted in 80% of the prison population being tested, including many housed in blocks that were not previously subject to surveillance testing. That testing resulted in identification of unrecognized transmission outside of quarantine wings, highlighting the limitations of surveillance strategies relying on close-contact and symptom-based testing, which may prolong outbreak duration (*28*). In addition, the availability of genomic evidence identified the recurring external introduction of SARS-CoV-2. Although the origins and pathways of the repeated introductions were unclear, vulnerabilities in entry quarantine procedures were revealed, and the importance of timely and extensive testing was reinforced. Continual widespread testing offers the advantage of detecting mild or asymptomatic infection in areas where clinical suspicion is low and provides the opportunity to monitor the effect of containment strategies. Identifying those gaps provides a framework for developing more robust prevention measures for SARS-CoV-2 and other infectious respiratory pathogens within congregate settings.

Housing location was a key contributor to infection acquisition. Persons in quarantine units experienced ≈2 times the risk for infection compared with those in general units. The increased risk for SARS-CoV-2 infection in quarantine areas was attributed, in part, to the placement of undetected community acquired cases with uninfected cellmates during their mandatory 14-day entry quarantine. However, before lockdown, cellmate-to-cellmate transmission accounted for only 10% of prison-acquired cases undergoing entry quarantine, indicating that other factors were more influential in transmission within dedicated quarantine wings. Although entry quarantine measures aim to confine potential transmission to quarantine wings, inadvertent exposure might occur. Specifically, interactions with mobile persons, such as incarcerated persons with special roles who move between areas, might unintentionally introduce infections into previously unaffected areas.

Cellmate exposure was associated with increased risk for infection. Although direct person-to-person transmission through close contact, whether between persons sharing a cell or direct interaction between incarcerated persons, appeared to be the primary mode of spread before the lockdown, transmission persisted even after movement restrictions were implemented. Phylogenetic clustering was observed among persons who were continuously housed alone or with an infected person for 14 days before their date of infection, supporting a shared transmission pathway despite the absence of known close contact. That clustering suggests close contact alone was not a prerequisite for SARS-CoV-2 transmission within a high-density congregate living environment and that the transmission of SARS-CoV-2 might be assisted by the structural and environmental characteristics inherent in prisons. This finding is necessary for planning preparedness and response to outbreaks of known and emerging airborne respiratory infections in such settings, particularly development of tailored interventions to mitigate transmission risk.

Although recent evidence has demonstrated vaccination and prior infection are major factors in reducing the infectiousness of index cases within prisons settings (*29*), data are limited on the effectiveness of mass-timed vaccination during SARS-CoV-2 outbreaks (*29*,*30*). Over the study period, the ongoing vaccination campaign progressively increased first dose coverage from 17% to 90% and second dose coverage from 4% to 64%. The result was a marked reduction in infection incidence with each additional dose administered. By using a time-varying Cox model that accounted for changing vaccination status throughout the study period, we found that, compared with unvaccinated persons, infection incidence was approximately half that among those who had received 1 dose and one quarter among those who received a second dose. That trend suggests a stepwise protective effect of vaccination against infections. Mass-timed vaccination should be considered a viable strategy for managing outbreaks of SARS-CoV-2 in prisons and congregate settings where other containment strategies may not be feasible, a finding that is relevant to other vaccine-preventable respiratory diseases.

Strengths of this study include complete capture of high-resolution spatiotemporal and person-day level data of the at-risk population, coupled with SARS-CoV-2 viral genomic information. Comparatively, in previously published studies, the absence of person-day level data required incidence rates and risk factors to be determined on the basis of a surrogate of the at-risk population size, typically the prison’s average population size or theoretical capacity (*3*,*4*,*7*). That limitation, combined with imprecise time-to-event data because of infrequent testing (*8*,*14*,*24*), can distort the accuracy and measurement of incidence and risk factor calculations. Although mass testing and symptom surveillance was conducted among staff, the level of detail available regarding daily staff movements and intra-prison contacts was insufficient to determine exposure risks or integrate into detailed chain-of-transmission analyses. However, available clinical and molecular epidemiology did not support transmission from staff to inmates, highlighting the predominance of inmate-to-inmate transmission. In addition, the Delta variant is no longer the globally predominant strain of SARS-CoV-2. Of consequence, variations in transmissibility, severity, and immune escape potential between SARS-CoV-2 variants might limit the generalizability of our results in settings or time periods involving other circulating variants.

Beyond the epidemiologic findings, a critical lesson from this study was the demonstrated potential of integrating data sources to develop standardized reporting systems for infectious diseases in enclosed congregate settings. In collecting person-day-level data that encompassed demographic, clinical, geospatial, and genomic information, we gained a deep understanding of the outbreak dynamic. Those data-driven insights emphasize the need for prisons to establish enhanced surveillance systems by using routinely collected datasets from both health and corrective services. Leveraging those data can enable timely decision-making and tailored interventions. To be effective, collaboration between health and corrective services is critical to ensure epidemiologic data are not only interpreted and integrated appropriately but also adapted to the unique operational nuances and demands of the correctional setting.

Our findings provide robust data on the factors associated with transmission of SARS-CoV-2 within prison settings and the feasibility of enhancing surveillance of infectious respiratory pathogens by using routinely collected data. This approach can be applied more broadly to guide the management of future respiratory infection outbreaks with epidemic potential in enclosed settings. During the initial stages of an outbreak, a timely and coordinated response is critical in limiting further spread and interrupting chains of transmission. When implemented together, strategic housing assignments, continual mass testing with rapid NAT, genomic sequencing, and mass timed vaccination can substantially reduce the risk for SARS-CoV-2 transmission and mitigate the severity of outbreaks in high-density living environments.

Appendix Additional information about surveillance of viral respiratory infections within maximum-security prison, Australia, 2021.
