# Supplementary material for: Surveillance of Viral Respiratory Infections within Maximum-Security Prison, Australia
Source: Emerg Infect Dis. 2025 Aug;31(8):1527–36. doi: 10.3201/eid3108.240571 (PMC12309748; doi:10.3201/eid3108.240571)
Supplement: Appendix — Additional information about surveillance of viral respiratory infections within maximum-security prison, Australia, 2021. [file 24-0571-Techapp-s1.pdf]

Article DOI: https://doi.org/10.3201/eid3108.240571

EID cannot ensure accessibility for supplementary materials supplied by authors. Readers who have difficulty accessing supplementary content should contact the authors for assistance.

Surveillance of Viral Respiratory Infections within Maximum-Security Prison, Australia

Appendix

Methods

This study followed the Strengthening the Reporting of Observational Studies in Epidemiology (STROBE) reporting guidelines for observational cohort studies.

Study design and setting

This was a prospective cohort study conducted in a prison in Sydney, Australia. The prison had a maximum operational capacity of 1,300 beds housing male remand (unsentenced), minimum- and maximum-security inmates. Structurally, the prison was divided into six housing units (Blocks A - F) and a clinic. Blocks A to E functioned as general housing for the prison population not in quarantine or isolation. Block F contained both general housing wings (wings 5 to 8) and four dedicated quarantine wings (wings 1 to 4) for people undergoing a mandatory 14-day quarantine period before entry (Appendix Figure 1). Block D, the minimum-security wing, was located outside the main prison walls. Operational housing capacity across Blocks ranged from 118 to 500 individuals, with an additional 30 beds in the clinic (Figure 1 in main text).

The study period spanned 48 days, commencing with the identification of the first COVID-19 case in the prison and ending on the date of the last laboratory-confirmed case.

Participants and data sources

Participants included all inmates housed in the prison during the study period (Appendix Figure 2). The study involved the analysis of routinely collected person-day-level data gathered by local health authorities and corrective services, and SARS-CoV-2 WGS data. These data combined custodial administrative information with electronic medical record data, including

sociodemographic characteristics, dates of prison entry and exit, dates of sample collection and results of SARS-CoV-2 nucleic acid testing (NAT), vaccination dose number and date administered, and housing location. Housing location data specified the block, wing, cell, and bed number of each person per-day. NAT was performed using the EasyScreen SARS-CoV-2 RT-PCR (Genetic Signatures) and/or the GeneXpert SARS-CoV-2 assay (Cepheid). Prison-wide surveillance SARS-CoV-2 testing, regardless of symptom or contact status, was undertaken by living unit on a continuous 72-hour basis.

Study definitions and outcomes

The date of infection was defined by symptom onset or positive NAT, whichever came first. The infectious period of positive cases was conservatively defined as starting 2 days before symptom onset or sample collection, whichever came first, and ending 14 days thereafter. Case definitions were assigned relative to their potential source of infection (community, prison, or unknown) and the confidence of this source of transmission (probable, possible, unknown) (Panel). Infection severity was defined clinically as asymptomatic, mild, moderate, or severe (*1*). The primary outcome was SARS-CoV-2 infection, defined as a positive SARS-CoV-2 NAT.

Whole genome sequencing

Samples with detectable SARS-CoV-2 RNA were sent to the Institute of Clinical Pathology and Medical Research, NSW Health Pathology for WGS to support contact tracing and cluster analysis. Viral genomes were extracted from upper respiratory tract swabs (typically nasopharyngeal) and PCR amplified using the Illumina Midnight sequencing protocol. PCR products were sequenced using the Illumina platform. A consensus sequence was generated from each sample to enable genomic sequence comparisons between suspected transmission clusters, using methods previously published (*2*).

The consensus genomes were aligned with MAFFT v7.471 (FFT-NS-2, progressive method) (*3*). The consensus genomes were manually inspected and any sequences missing >20% of the genome were excluded. Poor sequence read coverage was observed across the region 21381 and 21683 (with reference to the Wuhan strain); so this region was removed from all alignments. A phylogenetic tree visualizing sequence similarity between different samples was constructed using the maximum likelihood approach (IQTree v1.6.7 [substitution model: GTR+F+I] with 1,000 bootstrap replicates) (*4*). Transmission clusters were genomically defined

on the basis of shared mutational profiles and by clustering on the phylogenetic tree (Appendix Figure 4). Sequence pairs or clusters sharing less than three mutations (i.e., zero to two) was considered genomic evidence in support of direct (or recent) transmission.

Statistical and molecular analyses

Descriptive analyses were conducted to describe the housing location, demographic, and clinical characteristics of the prison population, and to assess the geographic distribution of SARS-CoV-2 infection. Plots visualizing the number of cases by positive specimen collection date and the 7-day average testing coverage over the study period were generated.

Incidence was calculated using person-time of observation and reported as the number of infections per 100 person-years (py). Confidence intervals (CI) for rates were calculated using a Poisson distribution. Time at-risk commenced on Day 1 of the study period, or the date of prison entry for individuals received later, and was censored at Day 48, or at the earliest occurrence of the individual testing NAT positive, being transferred out, or being released to freedom.

Cox proportional hazards regression analysis was used to estimate hazard ratios (HR) and 95% CIs to evaluate factors associated with SARS-CoV-2 transmission, using person-level time-varying covariates for changes in factors related to exposure. These factors were determined a priori and included housing location (general wing, quarantine wing), vaccination status (unvaccinated, 1-dose, and 2-doses), and cellmate exposure in the preceding 14 days. The frequency of cellmate exposures was determined over the course of the outbreak using a moving 14-day window to capture potential housing with a cellmate in their infectious period. These cellmate exposures were determined for all 1,562 study participants and categorized into three groups: housed alone, housed with a COVID-19 NAT negative cellmate, and housed with a COVID-19 NAT positive cellmate. Cumulative probability of infection was plotted according to the Kaplan–Meier method, and groups were compared using the log-rank test.

Movement of all positive cases and their cellmates between blocks, wings, and cells were mapped, and probable or possible chains of transmission, including the direction of transmission to the individual level were generated in combination with the case definitions (Panel). Genomic sequencing was available for 128 cases and was used to validate the hypothesized chains of transmission based on the epidemiologic data.

Analysis was performed using STATA 17 (version 17.0; Stata Corporation, College Station, TX). Data visualization was performed using Microsoft Power Bi (version 2.1) and Lucidchart.

Study oversight

As a public health priority, this outbreak investigation was conducted under the Public Health Act at the request of the NSW Ministry of Health, and in collaboration with St Vincent's Correctional Health NSW, Justice Health and Forensic Mental Health Network, the Institute of Clinical Pathology and Medical Research, and the Kirby Institute for Infection and Immunity in Society. Ethical approval was granted by the University of New South Wales (Sydney) Human Research Ethics Committee (HC220683).

Case definitions for potential location of infection acquisition and transmission source

Community Acquisition

- Incarcerated ≤ 14 days;
- Positive SARS-CoV-2 NAT on Day 1 of incarceration or within 14 days, and:
  - No close contact with a COVID-19 case in prison, or
  - Close contact in prison but differing genomic sequences (different sequence clusters).

Prison Acquisition

- Probable Source (Incarcerated > 14 days):
  – Positive SARS-CoV-2 NAT;
  – Close contact with a COVID-19 case in prison (e.g., cellmate, sweeper) or residing in the same wing within 14 days before infection, with or without supportive viral genomic evidence (same sequence cluster).
- Probable Source (Incarcerated > 48 hours and ≤ 14 days):
  – Negative SARS-CoV-2 NAT on entry;
  – Positive SARS-CoV-2 NAT within 14 days of incarceration;

– Close contact with a COVID-19 case in prison or residing in the same wing within 14 days before infection, with or without supportive viral genomic evidence.

- Possible Source:

– Incarcerated > 14 days;

– Positive SARS-CoV-2 NAT;

– Potential for close contact in the same wing, or brief face-to-face contact not meeting "close contact" criteria, within 14 days before infection, with supportive viral genomic evidence (same sequence cluster).

- Unknown Source

– Incarcerated > 14 days;

– Positive SARS-CoV-2 NAT;

– No epidemiological or genomic evidence identifying a probable or possible source.

References

1. National Institutes of Health. Clinical spectrum of SARS-CoV-2 infection. 2023 [cited 2023 Nov 5]. https://www.covid19treatmentguidelines.nih.gov/overview/clinical-spectrum

2. Rockett R, Basile K, Maddocks S, Fong W, Agius JE, Johnson-Mackinnon J, et al. Resistance mutations in SARS-CoV-2 Delta variant after sotrovimab use. N Engl J Med. 2022;386:1477–9. PubMed https://doi.org/10.1056/NEJMc2120219

3. Katoh K, Standley DM. MAFFT multiple sequence alignment software version 7: improvements in performance and usability. Mol Biol Evol. 2013;30:772–80. PubMed https://doi.org/10.1093/molbev/mst010

4. Nguyen LT, Schmidt HA, von Haeseler A, Minh BQ. IQ-TREE: a fast and effective stochastic algorithm for estimating maximum-likelihood phylogenies. Mol Biol Evol. 2015;32:268–74. PubMed https://doi.org/10.1093/molbev/msu300

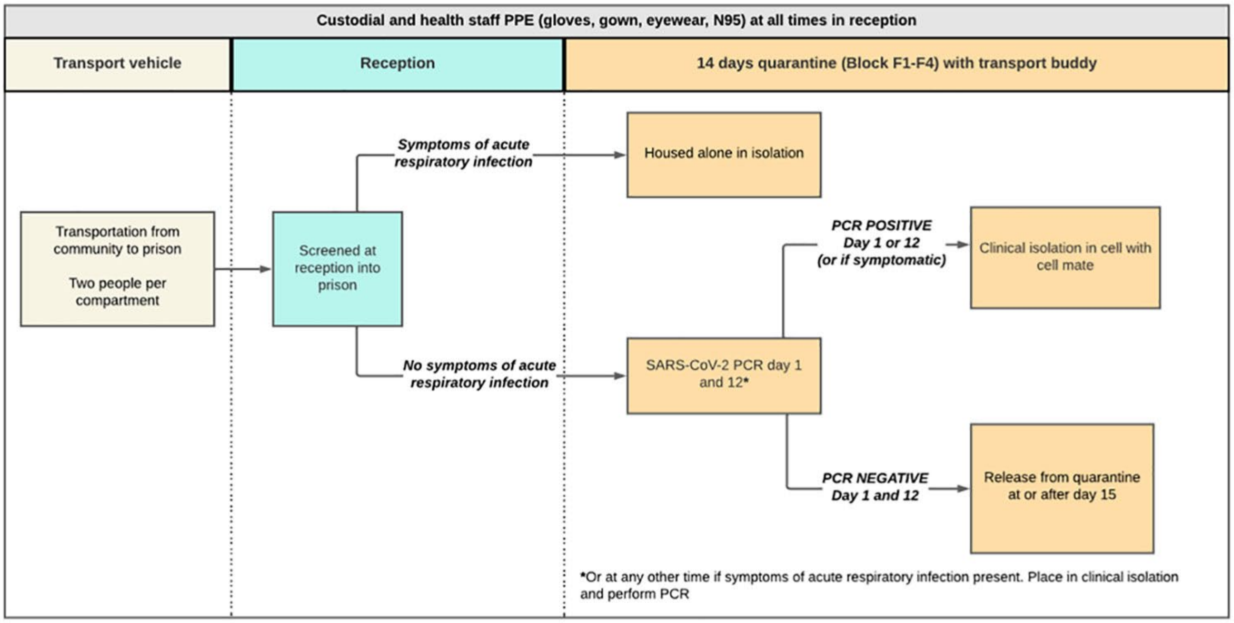

Appendix Figure 1. Entry screening process into prison pre-outbreak.

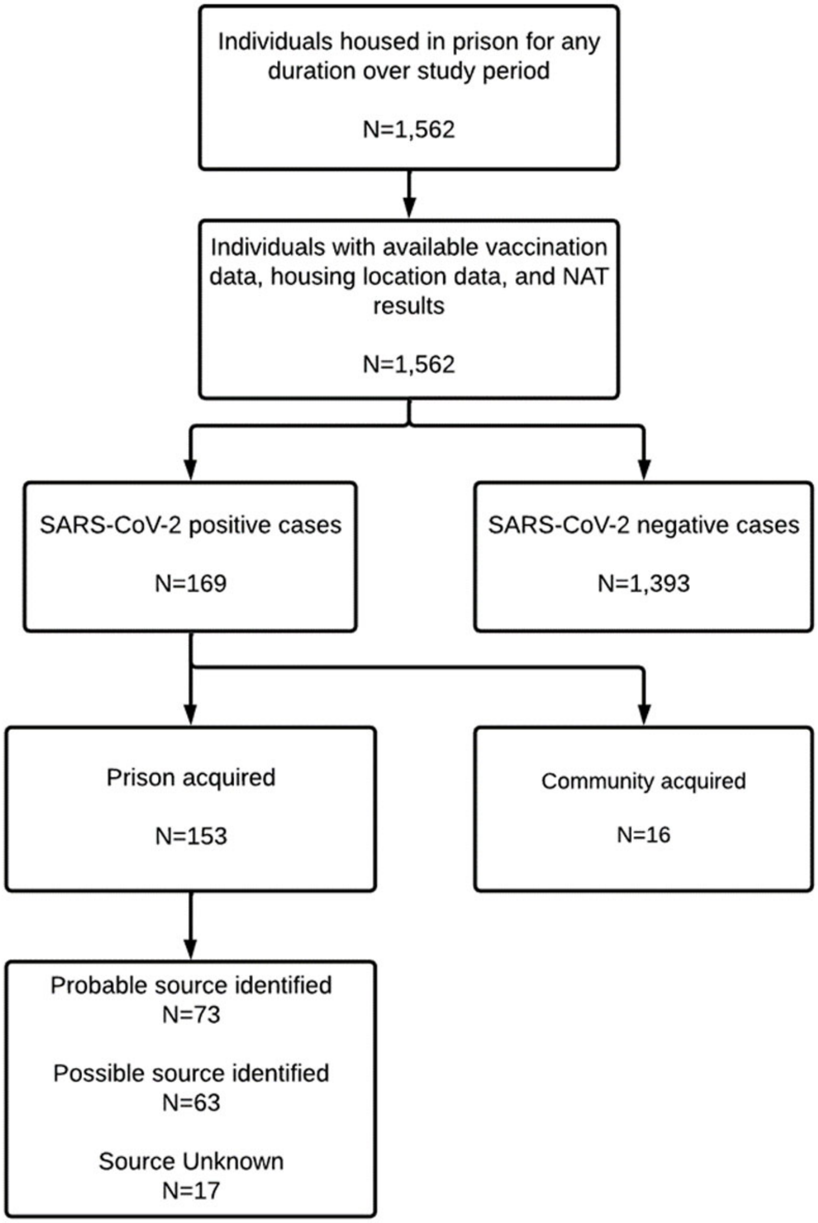

Appendix Figure 2. Overview of study population.

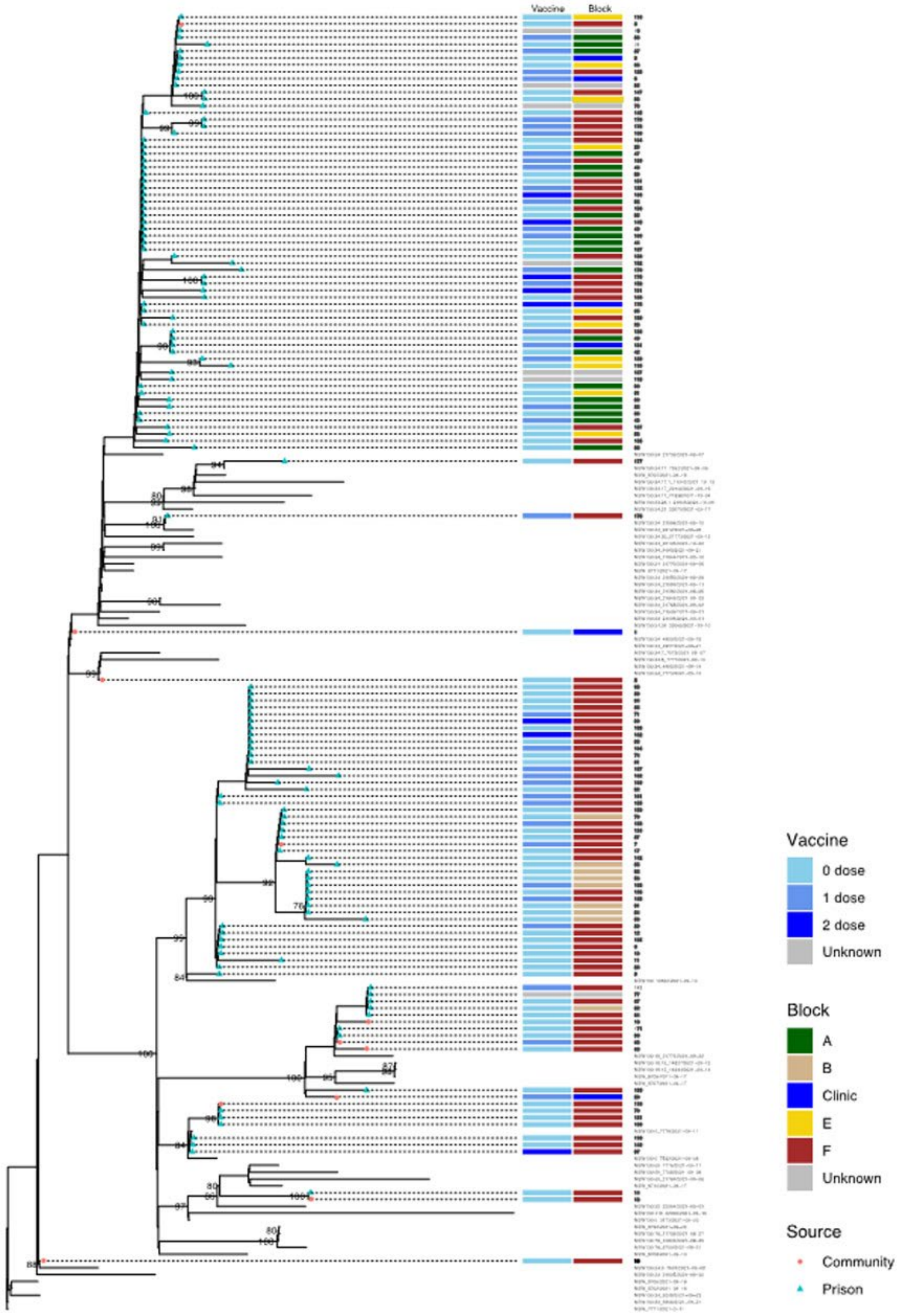

Appendix Figure 3. Phylogenetic analysis of near full-length SARS-CoV-2 genomes. Sequences isolated from the outbreak are numbered with their subject identifier. Additional sequences circulating in the NSW community were retrieved from GISAID and were added to the phylogeny. These are labeled with their NSW cluster ID followed by their GISAID number and date of collection. The percent bootstrap values in which the major groups were observed among 1000 replicates are indicated. The phylogenetic tree is unrooted, and the scale bar denotes the number of nucleotide substitutions per site.

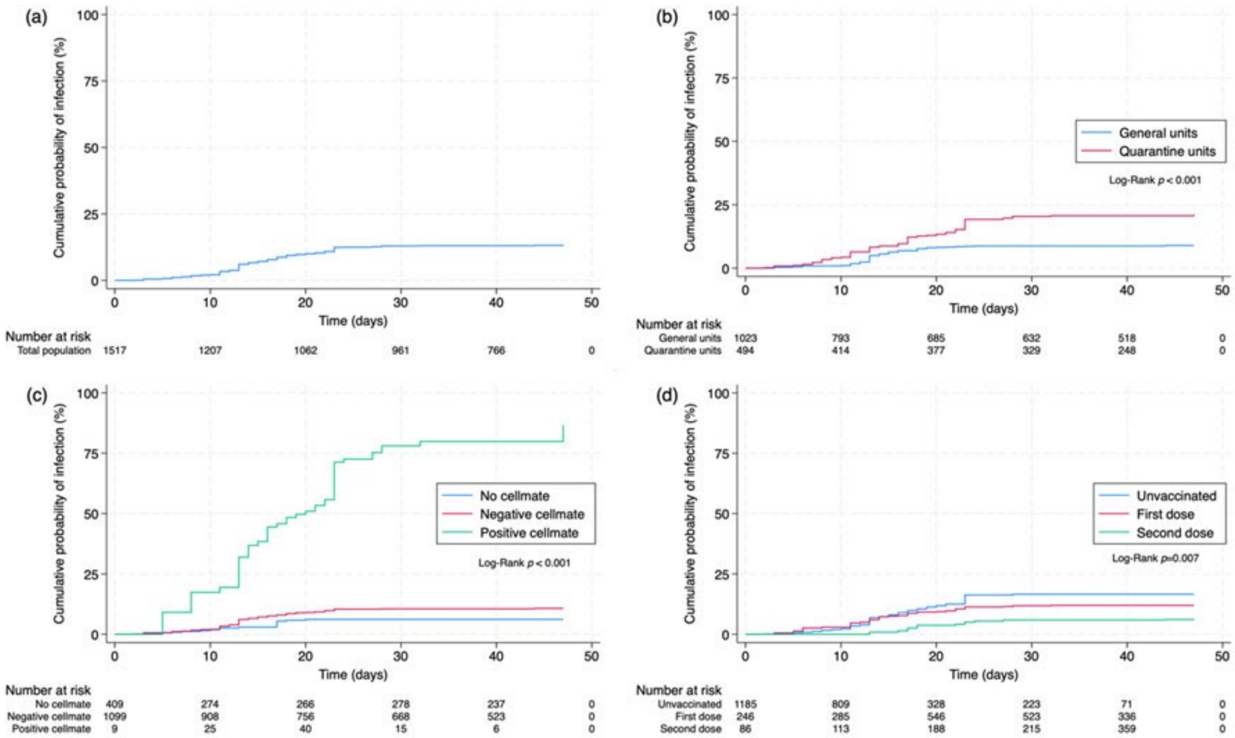

Appendix Figure 4. Cumulative probability of SARS-CoV-2 infection by: (a) overall population, (b) housing location, (c) cellmate status, and (d) vaccination status.

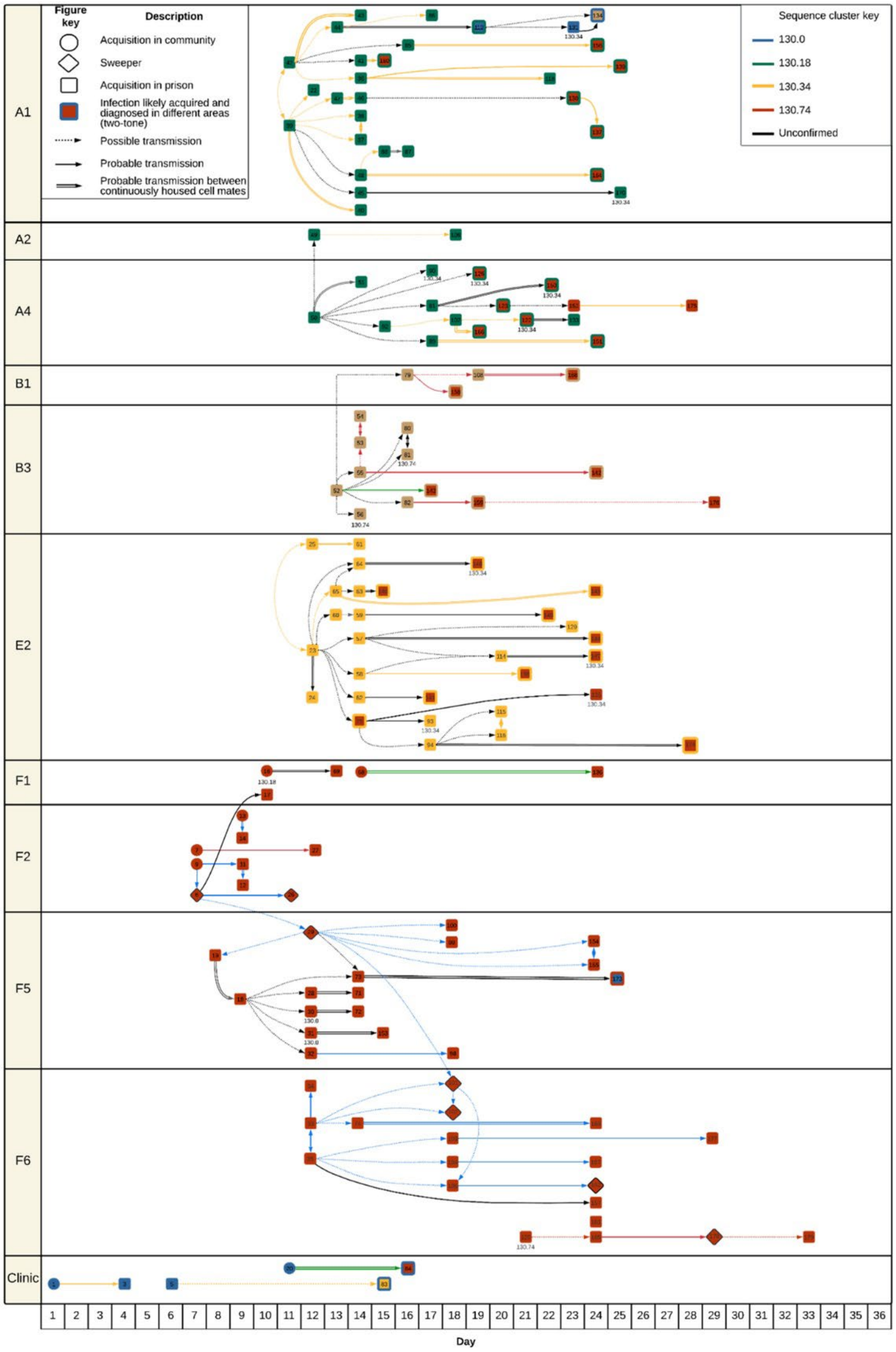

Appendix Figure 5. Probable and possible chains of transmission.
